# Supplementary figures and images for: Estimating the impact of mobility patterns on COVID-19 infection rates in 11 European countries
Source: PeerJ. 2020 Sep 15;8:e9879. doi: 10.7717/peerj.9879 (PMC7500353; doi:10.7717/peerj.9879)

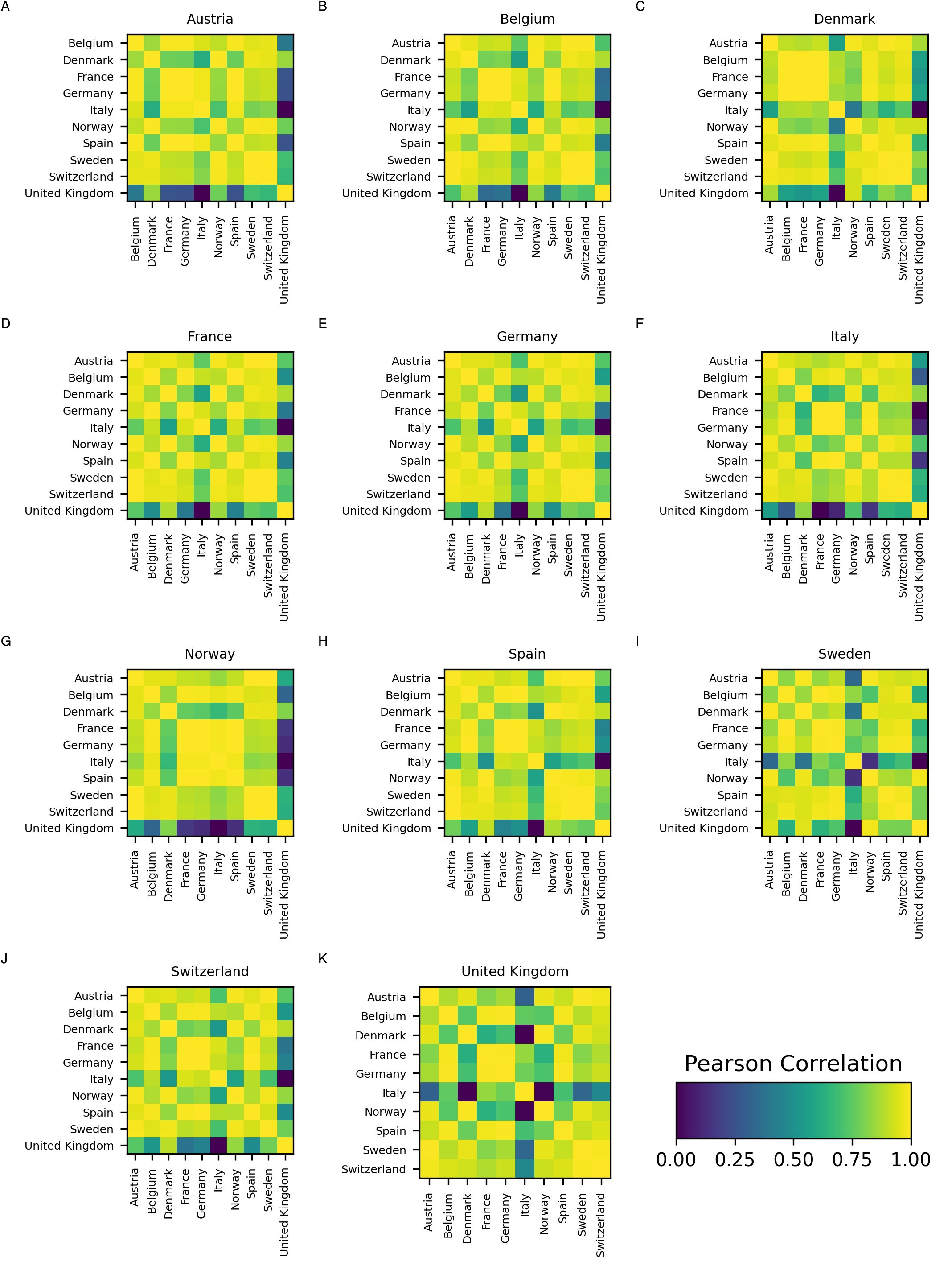

Supplement: Supplemental Information 1 — Visualization of the Pearson correlation coefficients for the mean R0 across all timepoints (including the forecast) for each country in the different runs when all other 10 (one per run) have been left out. [file peerj-08-9879-s001.png]

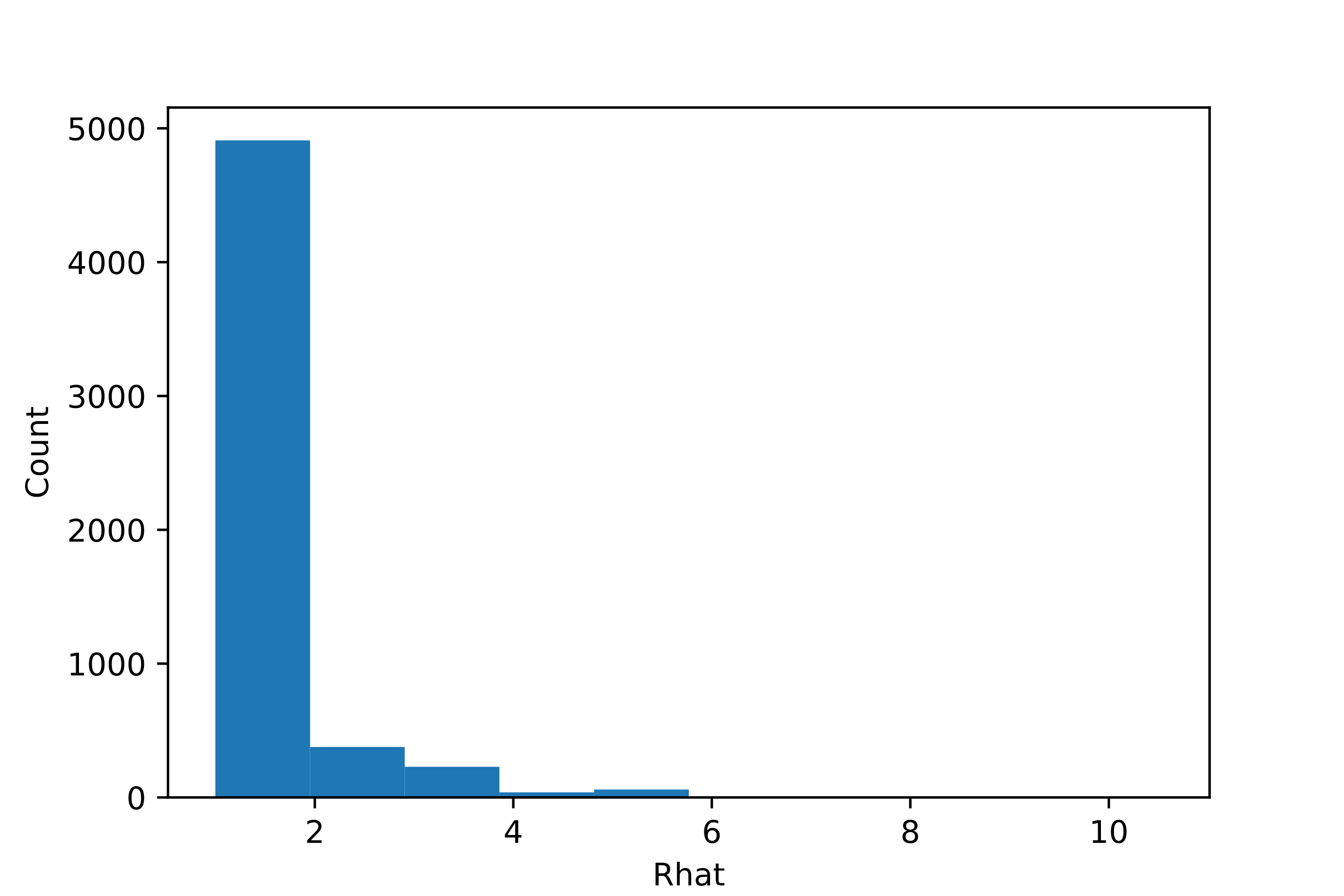

Supplement: Supplemental Information 2 — Values of 1 indicate convergence in the simulations. [file peerj-08-9879-s002.png]

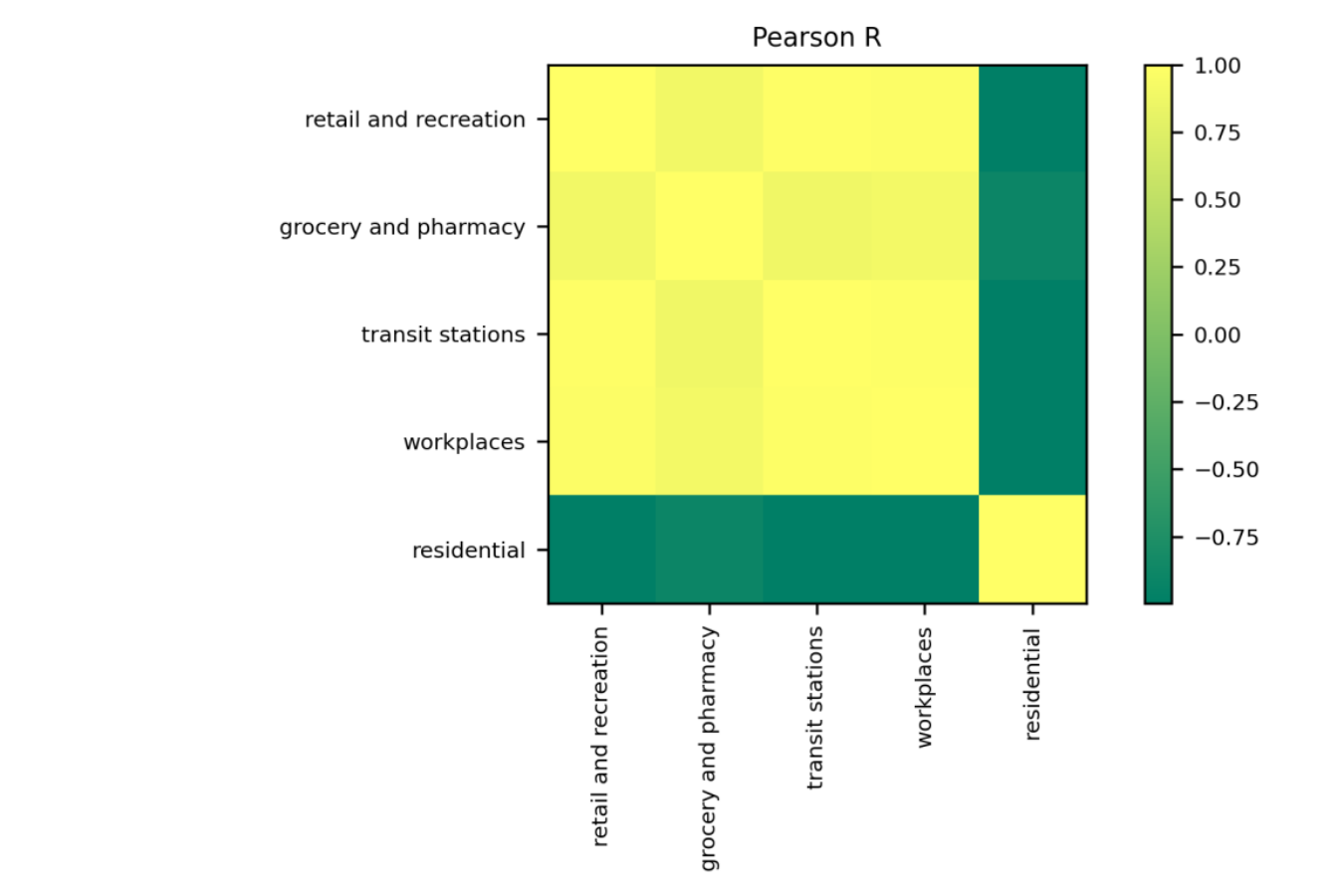

Supplement: Supplemental Information 3 [file peerj-08-9879-s003.png]

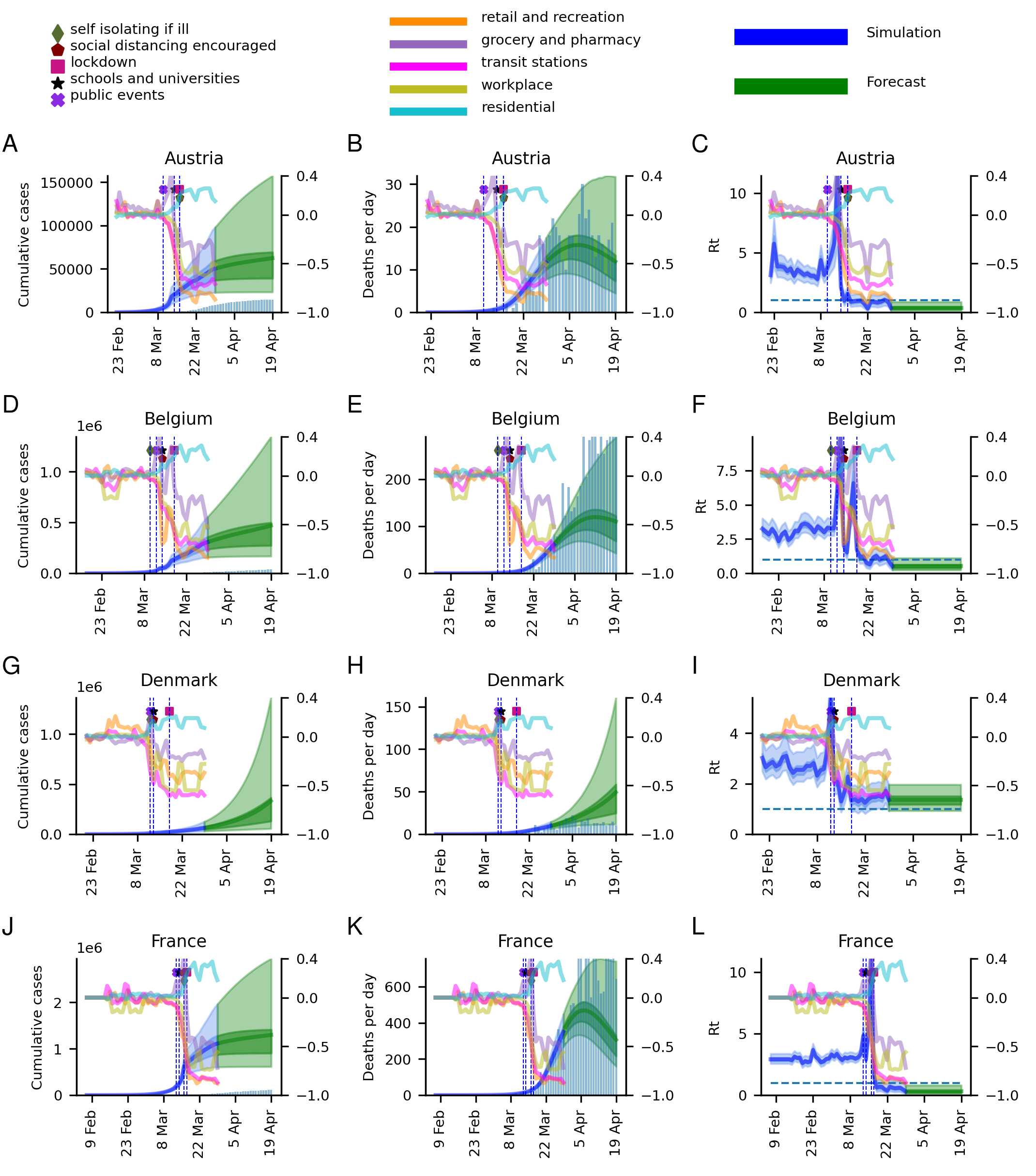

Supplement: Supplemental Information 4 [file peerj-08-9879-s004.png]

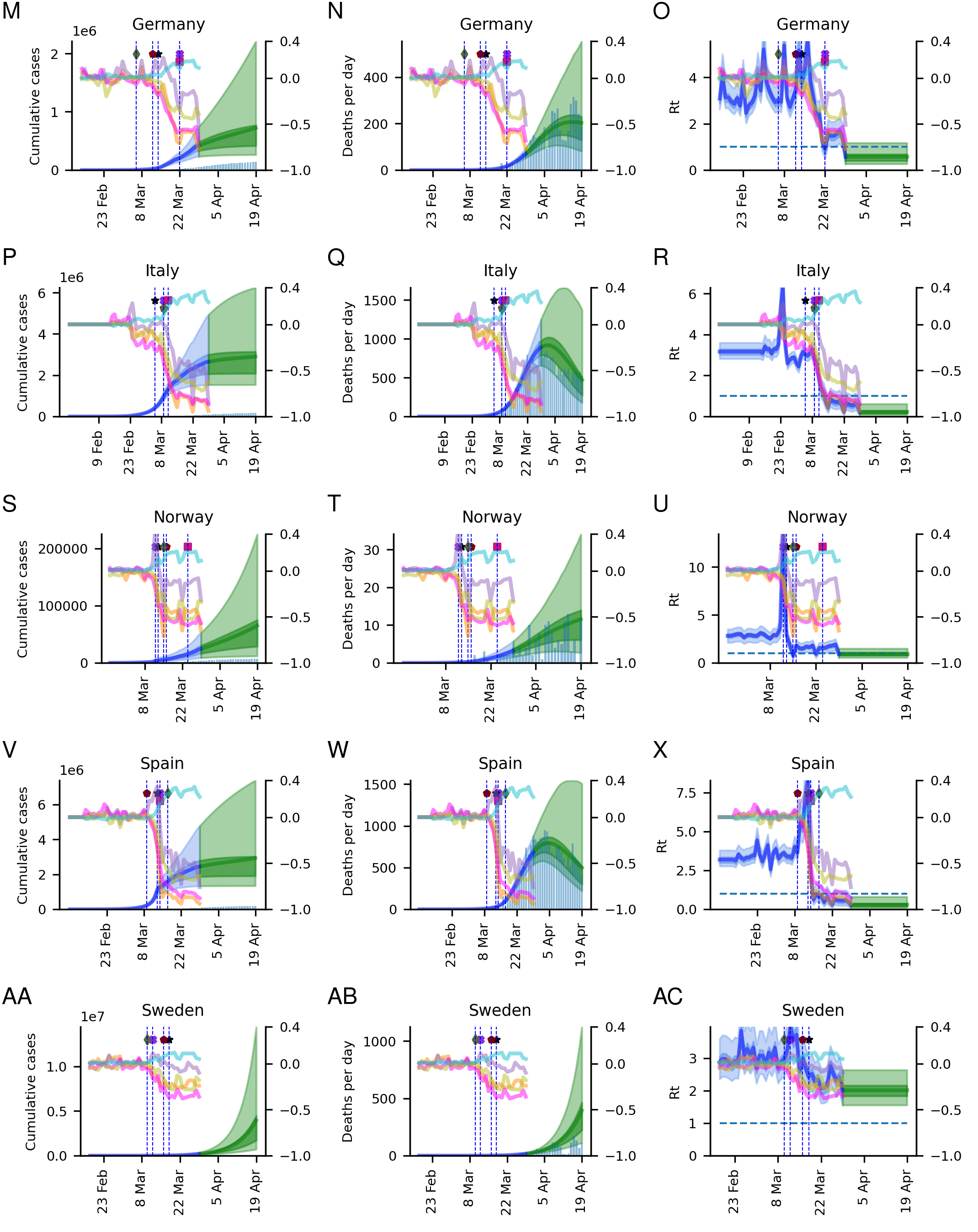

Supplement: Supplemental Information 5 [file peerj-08-9879-s005.png]

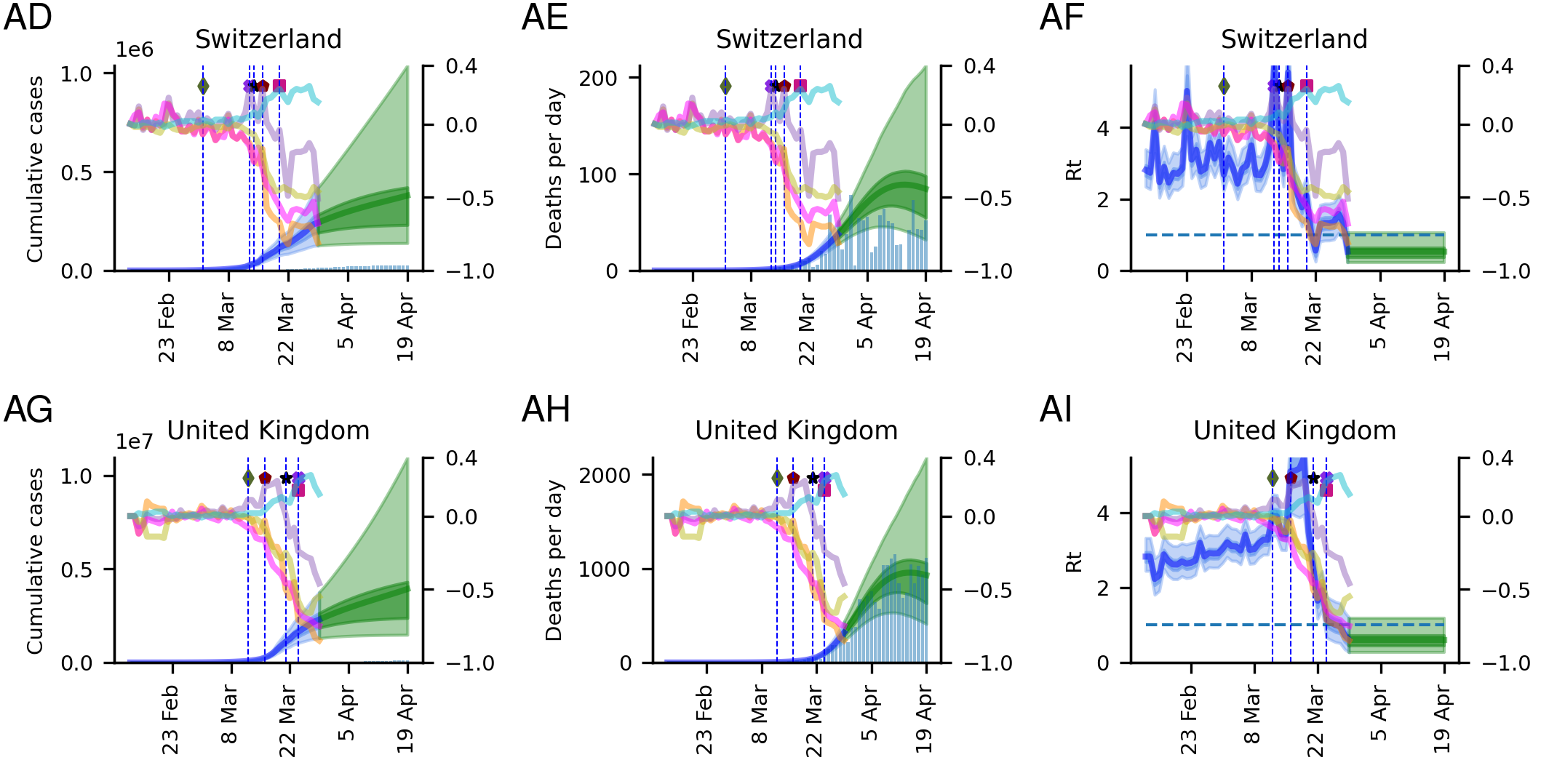

Supplement: Supplemental Information 6 — Model results in the form of cumulative number of cases, deaths per day and R0 for each respective country, are displayed on the left axes. The model results start from 30 days before 10 accumulated deaths had been observed. The blue curves represent the estimations so far, while the green represents a three-week forecast (30 March-19 April). The 50% and 95% confidence intervals are displayed in darker and lighter shades respectively, with the mean as a solid line. The histograms represent the number of cases and deaths reported by the European Center for Disease Control (ECDC). Mobility data for the five modelled sectors represented in terms of relative change compared to baseline (observed in a five-week period of 2020-01-03 to 2020-02-06) is displayed on the right axes. The dates for the introduction of different NPIs are marked with vertical lines. As can be seen, the NPIs have very strong implications for the mobility patterns. The mobility data ranges from 2020-02-15 to 2020-03-29, after which the final levels are fixed. The graph for Rt includes a dashed line marking the value 1 of halted epidemic growth. [file peerj-08-9879-s006.png]

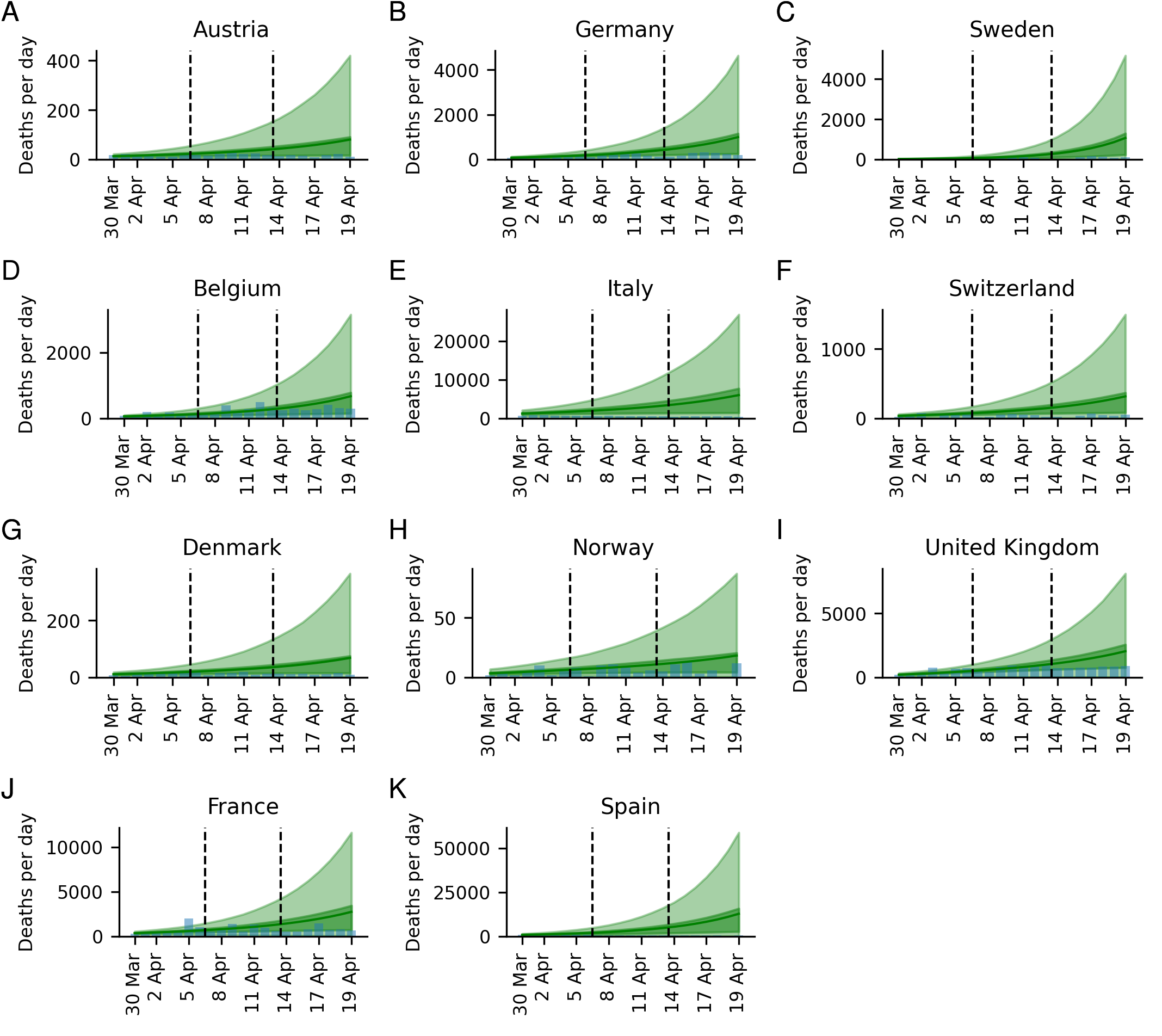

Supplement: Supplemental Information 7 — ICL model. Three week predictions for all countries in the form of deaths per day for the weeks Mar 30–April 5, April 6–April 12 and April 13–April 19. The 50% and 95% confidence intervals are displayed in darker and lighter shades respectively, with the mean as a solid line. The blue histogram represents the observed values. [file peerj-08-9879-s007.png]

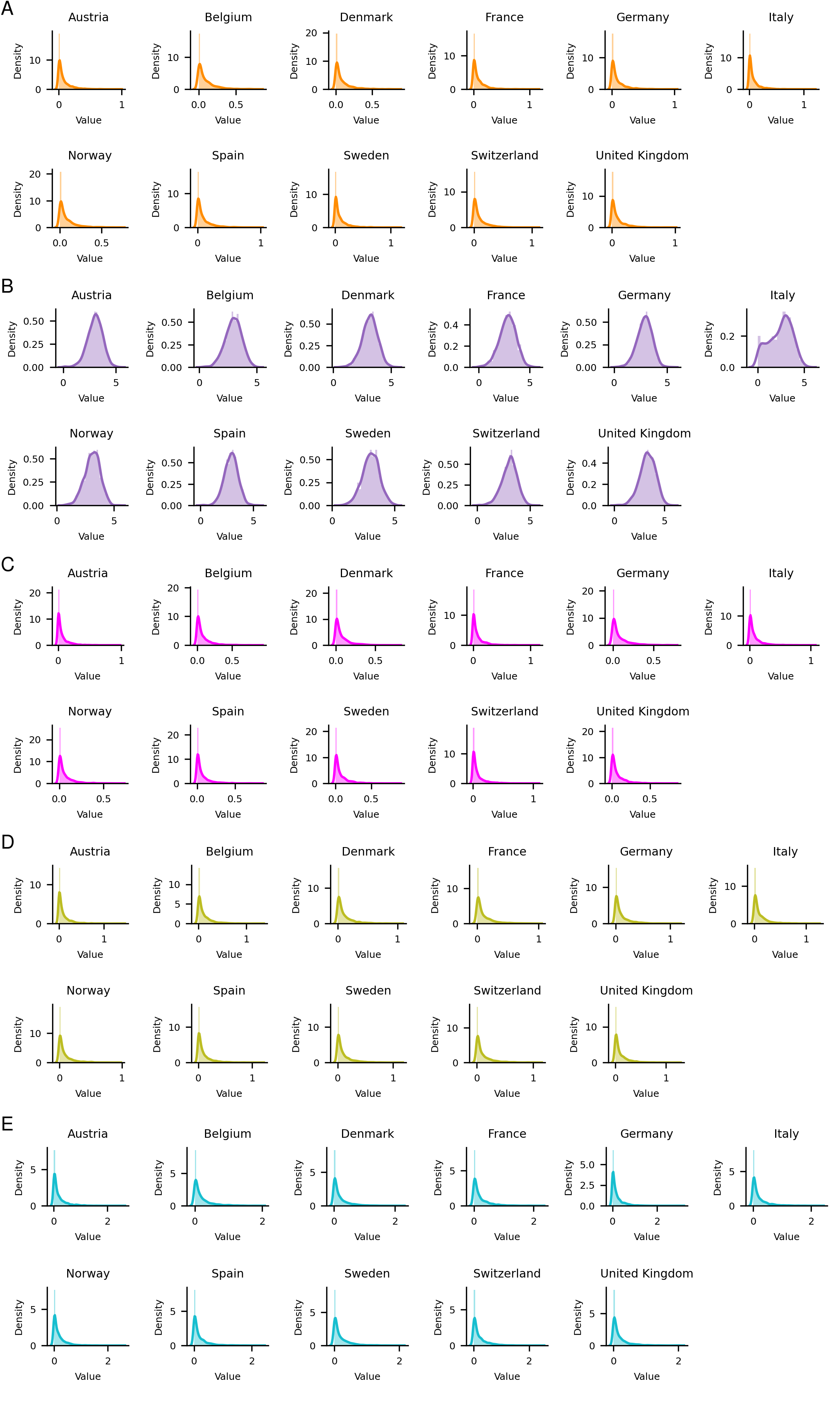

Supplement: Supplemental Information 8 — The order of the mobility sectors is retail and recreation (red), grocery and pharmacy (purple), transit stations (pink), workplace (olive) and residential (cyan). The titles of each distribution marks which country has been left out. The distributions are almost identical except for that of Italy in the grocery and pharmacy sector displaying more of a bimodality. The grocery and pharmacy sector appears to be the clearest indicator for R0 change. [file peerj-08-9879-s008.png]

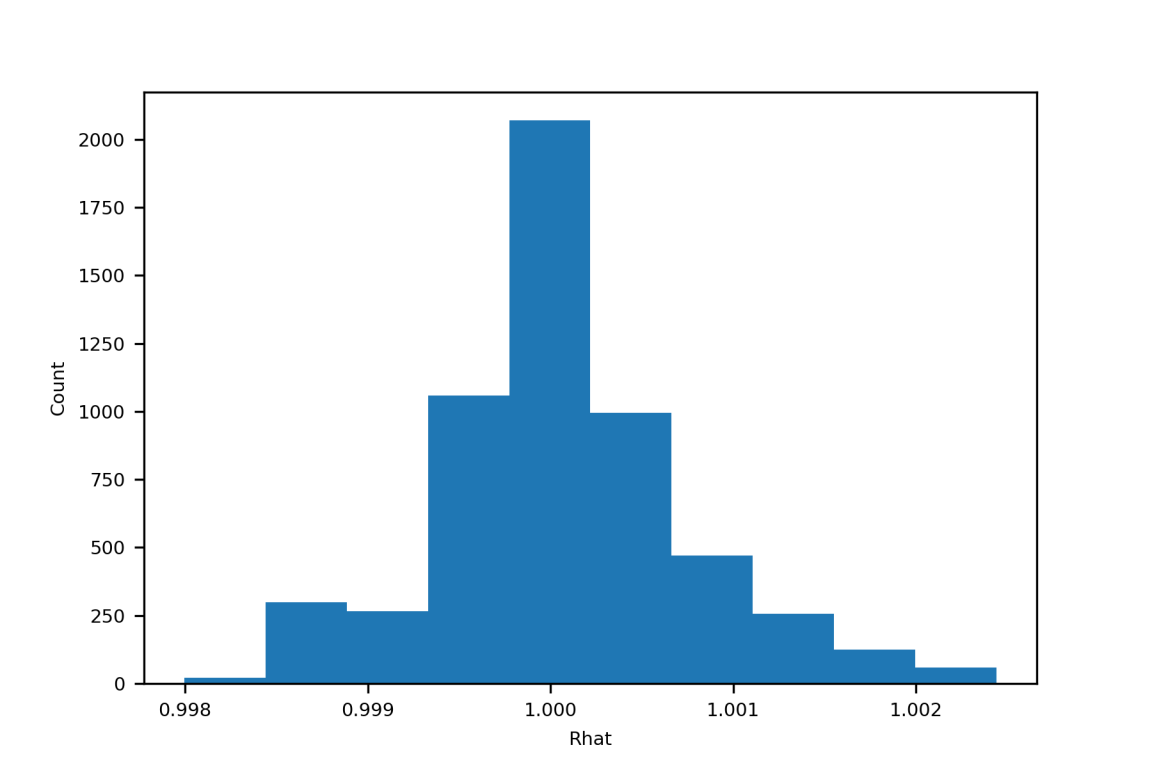

Supplement: Supplemental Information 9 — Values of 1 indicate convergence in the simulations. [file peerj-08-9879-s009.png]

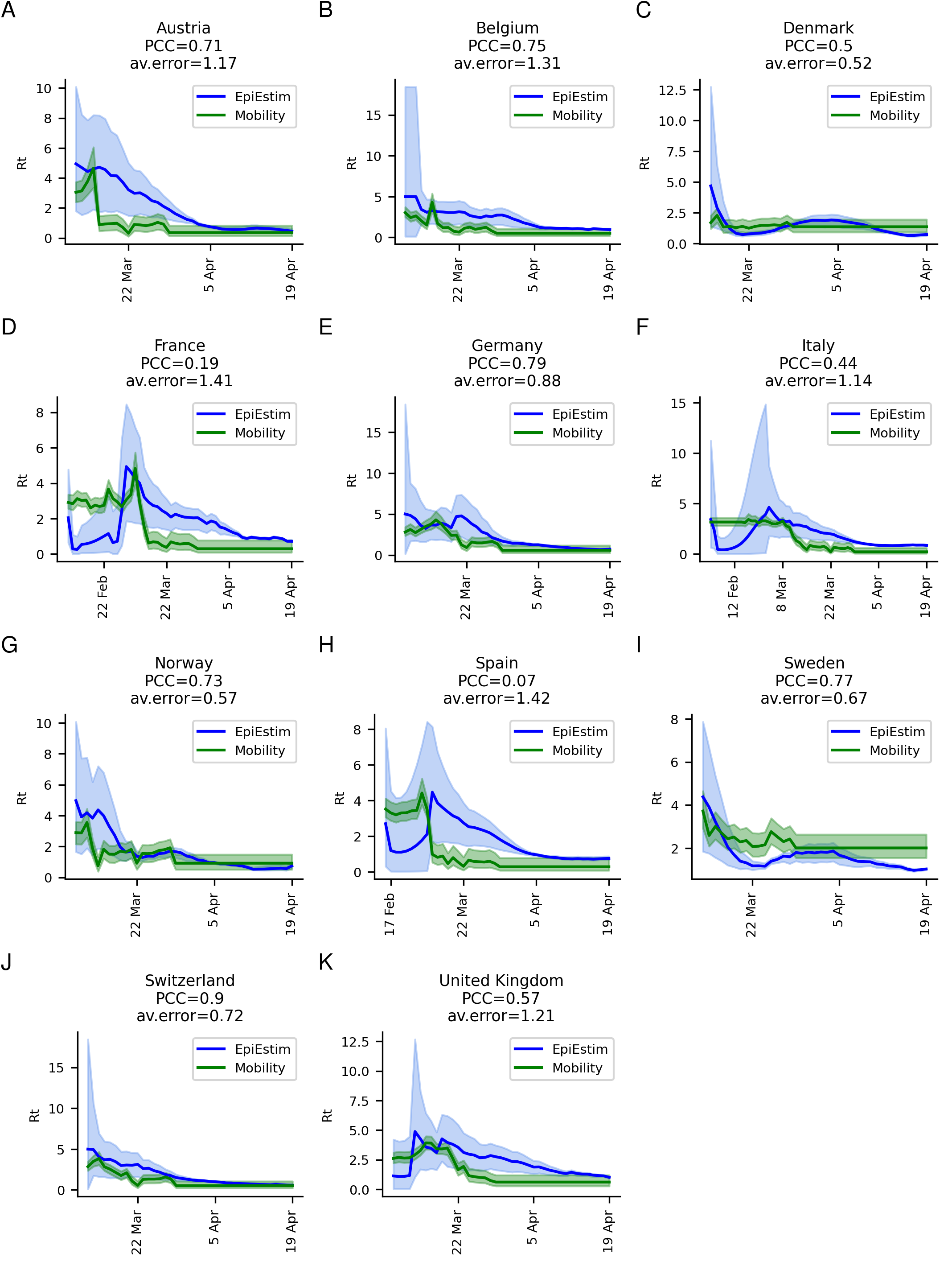

Supplement: Supplemental Information 10 — Only mean values below 5 are considered. The thick lines are the mean estimates while the shaded areas represent 95% confidence intervals. There are very large overlaps and the estimates correlate well. [file peerj-08-9879-s010.png]
