# Supplementary material for: Estimating the impact of mobility patterns on COVID-19 infection rates in 11 European countries
Source: PeerJ. 2020 Sep 15;8:e9879. doi: 10.7717/peerj.9879 (PMC7500353; doi:10.7717/peerj.9879)
Supplement: Supplemental Information 11 — Results for both our model (Mobility model) and the model from the Imperial College London team (ICL model) are shown. [file peerj-08-9879-s011.docx]

| **Comparison of three-week predictions between the Mobility and the ICL model** | | | | | | | | | | | | |
| --- | --- | --- | --- | --- | --- | --- | --- | --- | --- | --- | --- | --- |
|  | **Error in predicted deaths** | | | | | | **Average fractional error [%]** | | | | | |
|  | **Mobility** | **ICL** | **Mobility** | **ICL** | **Mobility** | **ICL** | **Mobility** | **ICL** | **Mobility** | **ICL** | **Mobility** | **ICL** |
| **Country** | **30 Mar - 5 Apr** | **30 Mar -5 Apr** | **6 Apr - 12 Apr** | **6 Apr - 12 Apr** | **13 Apr -19 Apr** | **13 Apr -19 Apr** | **30 Mar -5 Apr** | **30 Mar -5 Apr** | **6 Apr - 12 Apr** | **6 Apr - 12 Apr** | **13 Apr -19 Apr** | **13 Apr -19 Apr** |
| Austria | −3 | 0 | −6 | 9 | −2 | 45 | −2.3% | 0.2% | −4.0% | 6.1% | −1.7% | 42.2% |
| Belgium | −46 | −42 | −179 | −95 | −186 | 169 | −5.0% | −4.5% | −8.7% | −4.6% | −8.8% | 8.0% |
| Denmark | 0 | 1 | 10 | 13 | 28 | 39 | 0.4% | 1.1% | 10.2% | 12.7% | 32.2% | 45.5% |
| France | −318 | −247 | −445 | 93 | −427 | 1243 | −6.1% | −4.7% | −7.1% | 1.5% | −7.8% | 22.6% |
| Germany | −21 | −16 | −7 | 97 | −26 | 462 | −2.2% | −1.7% | −0.5% | 7.3% | −1.6% | 28.5% |
| Italy | 144 | 796 | 201 | 2042 | 29 | 4152 | 2.7% | 14.9% | 4.9% | 49.7% | 0.8% | 110.5% |
| Norway | 1 | 0 | 1 | 2 | 3 | 7 | 1.7% | 1.4% | 2.2% | 3.3% | 6.9% | 14.7% |
| Spain | −98 | 501 | 84 | 2653 | −8 | 8147 | −1.6% | 8.3% | 1.8% | 57.6% | −0.2% | 220.8% |
| Sweden | −3 | −1 | 28 | 71 | 180 | 527 | −1.2% | −0.5% | 5.4% | 13.8% | 28.9% | 84.4% |
| Switzerland | 13 | 9 | 41 | 68 | 48 | 191 | 4.3% | 3.1% | 14.1% | 23.4% | 17.1% | 68.6% |
| United Kingdom | 17 | −135 | −42 | −45 | 32 | 732 | 0.4% | −4.1% | −0.7% | −0.8% | 0.5% | 13.1% |
| Total absolute average | 60 | 159 | 95 | 472 | 88 | 1429 | 2.5% | 4.0% | 5.4% | 16.4% | 9.7% | 59.9% |
