# Supplementary material for: Estimating the impact of mobility patterns on COVID-19 infection rates in 11 European countries
Source: PeerJ. 2020 Sep 15;8:e9879. doi: 10.7717/peerj.9879 (PMC7500353; doi:10.7717/peerj.9879)
Supplement: Supplemental Information 12 — The dates compared are the date closest ahead in time after the first intervention with available values (Date 1) and the last modelled date (Date 2). [file peerj-08-9879-s012.docx]

| **Comparison of R estimates between the Mobility model and EpiEstim at the first intervention date and the last modeled date** | | | | | | | | |
| --- | --- | --- | --- | --- | --- | --- | --- | --- |
| **Country** | **Date 1** | **EpiEstim** | **Mobility** | **Difference 1** | **Date 2** | **EpiEstim** | **Mobility** | **Difference 2** |
| Austria | 2020-03-10 | 4.43 | 3.77 | 0.66 | 2020-04-19 | 0.45 | 0.36 | 0.09 |
| Belgium | 2020-03-14 | 3.42 | 2.03 | 1.39 | 2020-04-19 | 0.95 | 0.51 | 0.44 |
| Denmark | 2020-03-16 | 4.68 | 1.69 | 2.98 | 2020-04-19 | 0.72 | 1.36 | 0.63 |
| France | 2020-03-14 | 4.26 | 4.83 | −0.58 | 2020-04-19 | 0.72 | 0.30 | 0.42 |
| Germany | 2020-03-09 | 4.84 | 3.12 | 1.72 | 2020-04-19 | 0.70 | 0.56 | 0.13 |
| Italy | 2020-03-05 | 4.16 | 3.00 | 1.16 | 2020-04-19 | 0.86 | 0.22 | 0.64 |
| Norway | 2020-03-14 | 3.83 | 1.90 | 1.94 | 2020-04-19 | 0.75 | 0.92 | 0.17 |
| Spain | 2020-03-16 | 4.47 | 0.99 | 3.48 | 2020-04-19 | 0.76 | 0.29 | 0.47 |
| Sweden | 2020-03-14 | 4.38 | 3.72 | 0.66 | 2020-04-19 | 1.03 | 2.01 | 0.98 |
| Switzerland | 2020-03-12 | 4.95 | 3.50 | 1.44 | 2020-04-19 | 0.57 | 0.53 | 0.04 |
| United Kingdom | 2020-03-12 | 3.57 | 3.92 | −0.35 | 2020-04-19 | 1.00 | 0.61 | 0.40 |
